# Lost in Transition: The Need for a Strategic Approach to Facilitate Job Market Integration of Internationally Educated Physicians through Alternative Careers

**DOI:** 10.3390/ijerph19063503

**Published:** 2022-03-16

**Authors:** Tanvir C. Turin, Nashit Chowdhury, Deidre Lake

**Affiliations:** 1 Department of Family Medicine, Cumming School of Medicine, University of Calgary, Calgary, AB T2N 4N1, Canada; nashit.chowdhury@ucalgary.ca; 2 Department of Community Health Sciences, Cumming School of Medicine, University of Calgary, Calgary, AB T2N 4N1, Canada; 3 The O’Brien Institute for Public Health, Cumming School of Medicine, University of Calgary, Calgary, AB T2N 4N1, Canada; 4 The Libin Cardiovascular Institute, Cumming School of Medicine, University of Calgary, Calgary, AB T2N 4N1, Canada; 5 The Newcomer Research Network, University of Calgary, Calgary, AB T2N 1N4, Canada; 6 Alberta International Medical Graduates Association, Calgary, AB T2E 3K8, Canada; deidre@aimga.ca

**Keywords:** job market integration, internationally trained physicians, international medical graduates, foreign medical graduates, health and wellness, transferable skills

## Abstract

Skilled migration has been an important part of the socioeconomic development and progression of many industrialised Western countries. However, successful migration includes facilitating sociocultural and professional environments, policies, and practices in a way that utilizes the skills of migrants appropriately. Internationally educated physicians (IEPs) are an important part of the health and wellness care program of these countries. Nevertheless, because of regulations and limited available positions, many of these migrated physicians find they cannot enter into the healthcare workforce as practicing physicians. Utilizing their health-related knowledge and skills through nonphysician careers in health and wellness is a beneficial way to integrate these highly skilled professionals into a country’s socioeconomic flow. Despite the availability of alternative careers for IEPs, we identified that these paths are often not explored and facilitated, resulting in un/underemployment and wastage of these highly skilled human resources. A lack of willingness among IEPs, under/overestimation of their transferable skills by themselves and by potential employers, and a lack of strategic support and career guidance are prominent obstacles. A collaborative approach from multiple sectors, including academics, integration service providers, and policy makers, is needed to create awareness of these alternative opportunities and facilitation of the socioeconomic integration of IEPs.

## 1. Introduction

Immigration is a process through which individuals from one country permanently move to another country [1]. Immigration has been a useful strategy for economic, social, and cultural development for many countries [1]. The immigrant population is often an excellent pool of human resources with diverse skills and expertise. Amongst these highly skilled immigrants are internationally educated physicians (IEP), international medical graduates, or foreign medical graduates “who gained their qualifications from a medical school in a country other than the one in which they seek to professionally integrate” [2,3]. In 2017/18, more than one in six doctors working in an Organisation for Economic Co-operation and Development (OECD) country, which includes Canada, the USA, the UK, Australia, New Zealand, and more, had obtained their medical degree in another country, up from one in seven a decade earlier [4]. According to reports, about 37% of physicians in the UK are not UK graduates [5,6], more than 40% of practicing doctors in New Zealand are trained overseas [7], and about one in three Australian doctors are foreign-trained [8]. In Canada and the US, the figure lies around 26% [9,10].

To become a licensed physician in their newly adopted host country, IEPs first need to clear the certification requirement by the appropriate local licensing authorities. The certification process, combined with the competitive nature of residency selection and strict licensure rules, ensure that only rigorously screened IEPs enter the physician workforce. While a proportion of practicing physicians in some countries are IEPs, many other IEPs are unable to practice as physicians in these countries and remain un/underemployed. For example, according to one report, about 74% of foreign-trained physicians in New Zealand who passed the mandatory licensing exam in the last two years were unable to find a job in medicine [11]. In the US, around 40–50% of IEPs cannot be matched to a residency spot, which is a crucial requirement for them to be eligible to practice as physicians [12]. The current situation in Canada is harsher, with around 80% or more of IEPs unable to secure a residency position to practice medicine as a physician [13,14].

These unlicensed IEPs repeatedly attempt to become licensed; however, only a handful of them succeed, leaving the rest with no choice but to seek other options [15,16]. Many, unfortunately, cannot find a decent alternative career and are forced to undertake jobs that do not require high skills to meet livelihood responsibilities [3,16]. As IEPs may never have considered a career apart from being a physician, when they encounter such a situation, they feel lost [13]. Most of the supports provided by newcomer resettlement organizations seem to focus on the licensing process. Given the circumstances, this article discusses ways to facilitate alternative career pathways that afford unlicensed IEPs the opportunity to transfer their medical knowledge and skills to non-physician health and wellness positions (often referred to as alternative careers for IEPs), with a view to optimize the employment of these highly skilled human resources and increase the economic productivity of the host country [17]. Alternative careers for some IEPs are a lifeline as they opt out of going through the rigour and uncertainty of the physician licensure pathway.

## 2. Alternative Career Pathways in the Health and Wellness Sector

**Figure 1:**
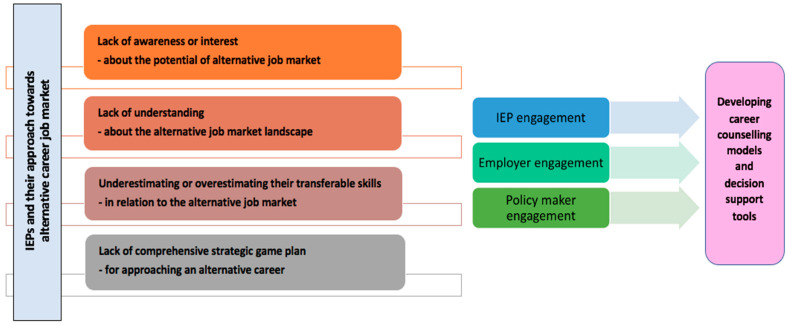
IEPs and alternative career approach issues. IEP: internationally educated physician.

Alternative careers allow IEPs to regain their professional identity, which may have been lost in the migration process [18]. For example, by becoming a health policy analyst, an IEP can utilize their knowledge of healthcare services and disease prevention, have excellent career prospects, and contribute to the healthcare system and economy of the host country. There are numerous impediments to the facilitation of IEPs into alternative careers. Challenges exist both at the IEP and system level, but IEP-level barriers seem to be the most challenging as well as the most comprehensively unexplored (Figure 1).

### 2.1. Lack of Interest by IEPs in Pursuing Alternative Career Pathways

A common phenomenon observed of IEPs is their lack of interest in pursuing alternative careers. One study showed that most IEPs generally have never considered seeking any career other than becoming a physician [19]. A study in Canada reported that the majority of IEPs move to Canada predominantly through the skilled migration process, believing that they were needed, with the aim of continuing their career as a physician [20]. If they knew they might need to seek an alternative career, perhaps they would have reconsidered their decision of migrating or they would migrate being mentally prepared for the need to pursue an alternative career [18,20]. This is also linked to another reported individual-level barrier—the delay in commencing an alternative career [16,21]. One study showed that IEPs who had joined a fast-tracked nursing program as an alternative career had unsuccessfully attempted to obtain US medical residency for at least five years before deciding to pursue the fast-track program [22]. Further, another study reported that IEPs who opted to seek alternative careers expressed regret over not making that decision earlier [16]. This delay can deprive IEPs of time that may be required to study for the new position and obtain any required health-related work experience as well as increase family responsibilities and financial burdens, all of which ultimately lead to a loss of energy and lack of money to spend following their training and education to allow for a decent alternative career [23,24].

### 2.2. Mismatch in Self-Assessment: Under- or Overestimating Transferable Skills

IEPs initially may not perceive the challenges involved in pursuing an alternative career; it will require perseverance, determination, and hard work to achieve a successful alternative career. The learning curve involved in seeking and securing employment can potentially decrease their interest in pursuing an alternative career [21]. Further, many IEPs migrate to the host country at a stage of their life when they are burdened with family responsibilities and in no position to take the time to train extensively for an alternative career [24]. A successful career pursuit requires a professional network. However, naturally, because of their physician background, most IEPs’ professional networks are generally limited to those in the physician/physician licensure realm [25]. Another important challenge is the lack of understanding of potential alternative careers and the transferable skills IEPs can utilize in those jobs [26]. This lack of knowledge obscures them from identifying an alternative career that suits their skills, aptitude, and individual perspectives (e.g., financial constraints, family responsibilities, etc.), which, in turn, hinders them from making an informed decision about the alternative career [17]. Sometimes, overestimating transferable skills and not expending the time and effort to polish those skills may affect their competitiveness as job candidates. For example, an IEP may believe that he/she has management skills because they ran a ward or department of a hospital in their home country and oversaw doctors, interns, nurses, and other healthcare professionals working under their management. However, the management role may be entirely different in the host country and require certain skill sets that are obtained by specific management-related education and training (e.g., project management, design thinking, program evaluation, etc.). While their previous management experience should be recognized, IEPs may need to undergo additional training/job shadowing to understand the context of the new management position before assuming that particular role.

### 2.3. Insufficiency in the Comprehensive Strategic Game Plan Required for Approaching an Alternative Career

The lack of a proper understanding about the job market and lack of a clear understanding of where they stand as job candidates also impacts IEPs’ preparation for any job application or interview [3]. Inadequately researching and exploring the specifics of an alternative job, failing to obtain the required training/volunteer experience for the job, applying for the job with an inappropriate resumé for the particular job, and being underprepared for interviews are all aspects of the job search process that have been observed to be negatively affecting the IEPs’ job search performance. Many IEPs continue to apply for jobs without a strategic plan, proper self-development, or the determination required to succeed [13].

## 3. Policy Recommendations: Developing a Systematic Career Counselling Model

As indicated above, IEPs may lack sufficient knowledge about alternative career options, including what the requirements for the careers are, what transferable skills they have for the career, how to prepare themselves, how to apply for jobs, and, depending on their skills and aptitude, what other career options might be available to them. Moreover, livelihood needs, family responsibilities, financial constraints, and other factors play important roles in the decision-making process. A strategic career counselling model and roadmap is recommended to help guide IEPs in choosing and preparing for alternative careers based on their transferable skills and other decision-making factors arising from personal preferences and constraints. Developing a comprehensive decision-making guide will not only directly help IEPs to pursue a suitable alternative career but will also help service providers to direct appropriate career pathways to newcomer IEPs. This will also help guide them to adequately prepare and develop an executable career plan for better professional integration.

## 4. Future Research Needs

The proposed strategic career counselling and coaching roadmap needs to be evidence-based, and several issues need to be explored systematically through research. This might involve obtaining feedback from both IEPs and stakeholders to find a common ground where IEPs can utilize their skills and knowledge, employers get high-quality service from them, and policy makers utilize IEPs’ diversity to serve vulnerable populations in the community, thereby increasing the economic contribution of the employed IEPs and more. Potential areas of research towards this end include:

(1) Inquiring into the needs, barriers, facilitators, and career goals of IEPs.
(2) Investigating IEPs’ awareness and perceptions of preparing for an alternative career in the health and wellness sector.
(3) Identifying what transferable skills and skill building IEPs require.
(4) Exploring different stakeholders’ (e.g., potential employers, policymakers, etc.) perceptions about IEPs as potential employees.

## 5. Conclusions

Many IEPs encounter extreme difficulties in becoming licensed physicians in the countries into which they have migrated. While alternative career pathways have the potential to reduce the un/underemployment generated by the unsuccessful pursuit of licensure, there is a lack of awareness, interest, and strategic support for these pathways. A collaborative and evidence-based approach is needed to develop an alternative career support strategy and inform policies to increase awareness among IEPs and stakeholders regarding alternative careers. It is imperative to work towards supporting IEPs to find and grow in suitable alternative careers. A systematic structure needs to be devised to identify, nurture, and polish the transferable skills of IEPs. The biggest challenge to supporting this concept is securing the buy-in of both IEPs and stakeholders towards a strategic approach to facilitating job market integration of IEPs into alternative careers.

## Data Availability

Not applicable.

## References

[1] (2019). Britannica. https://www.britannica.com/topic/immigration.

[2] (2019). Educational Commission for Foreign Medical Graduates. https://www.ecfmg.org/certification/definition-img.html.

[3] Turin T.C., Chowdhury N., Ekpekurede M., Lake D., Lasker M.A.A., O’Brien M., Goopy S. (2021). Professional integration of immigrant medical professionals through alternative career pathways: An Internet scan to synthesize the current landscape. Hum. Resour. Health.

[4] Socha-Dietrich K., Dumont J. (2021). International Migration and Movement of Doctors to and within OECD Countries—2000 to 2018: Developments in Countries of Destination and Impact on Countries of Origin.

[5] Jalal M., Bardhan K.D., Sanders D., Illing J. (2019). Overseas doctors of the NHS: Migration, transition, challenges and towards resolution. Future Healthc. J..

[6] (2015). General Medical Council. http://www.gmc-uk.org/doctors/register/search_stats.asp.

[7] Auckland District Health Board. https://www.adhb.health.nz/health-professionals/careermed/specialty-information-2/specialty-information-3/.

[8] Health Voices (2016). Journal of the Consumers Health Forum of Australia. http://healthvoices.org.au/issues/november-2016/health-workforce-whos-global-strategy-to-meet-a-global-challenge/.

[9] Ranasinghe P.D. (2015). International medical graduates in the US physician workforce. J. Am. Osteopath. Assoc..

[10] (2020). Canadian Institute for Health Information (CIHI). https://secure.cihi.ca/free_products/physicians-in-Canada-report-en.pdf.

[11] (2018). Stuff. https://www.stuff.co.nz/national/health/103932802/foreign-doctors-struggling-to-get-jobs-in-new-zealand.

[12] (2020). National Resident Matching Program. https://www.nrmp.org/wp-content/uploads/2020/06/MM_Results_and-Data_2020-1.pdf.

[13] Alberta International Medical Graduates Association (AIMGA) (2020). Career Transition Program for IMGs An Exploration of Alternative Pathways into Healthcare.

[14] (2019). Canadian Resident Matching Service. https://www.carms.ca/pdfs/2019-CaRMS-Forum.pdf.

[15] (2016). STAT. https://www.statnews.com/2016/11/28/residency-failed-to-match/comment-page-3/.

[16] Blain M.J., Fortin S., Alvarez F. (2017). Professional journeys of international medical graduates in Quebec: Recognition, uphill battles, or career change. J. Int. Migr. Integr..

[17] (2013). Lim Consulting Associates. https://novascotia.ca/lae/RplLabourMobility/documents/AlternativeCareersResearchReport.pdf.

[18] Shuval J.T. (2000). The Reconstruction of Professional Identity among Immigrant Physicians in Three Societies. J. Immigr. Health.

[19] Beran T.N., Violato E., Faremo S., Violato C., Watt D., Lake D. (2012). Ego identity development in physicians: A cross-cultural comparison using a mixed method approach. BMC Res. Notes.

[20] Moneypenny C.R. (2018). Understanding the Experiences of International Medical Graduates (IMGs) in Ontario, Canada: A Qualitative Study. Master of Science Thesis.

[21] (2019). SEAK. https://seak.com/the-biggest-mistakes-physicians-make-in-transitioning-to-a-non-clinical-career-and-how-to-avoid-them/.

[22] Flowers M., Olenick M. (2014). Transitioning from physician to nurse practitioner. J. Multidiscip. Healthc..

[23] Chowdhury N., Ekpekurede M., Lake D., Chowdhury T.T. (2021). The alternative career pathways for international medical graduates in health and wellness sector. International Medical Graduates in the United States.

[24] (2017). University of Toronto. https://tspace.library.utoronto.ca/handle/1807/79478.

[25] Community Matters Toronto. n.d. http://communitymatterstoronto.org/wp-content/uploads/2017/07/Alternative-Careers-for-IEHPs-.pdf.

[26] Turin T.C., Chowdhury N., Ekpekurede M., Lake D., Lasker M.A.A., O’Brien M., Goopy S. (2021). Alternative career pathways for International Medical Graduates towards job market integration: A literature review. Int. J. Med. Educ..

